# Understanding Practical, Robust Implementation and Sustainability of Home-based Comprehensive Sexual Health Care: A Realist Review

**DOI:** 10.1007/s10461-024-04415-x

**Published:** 2024-07-04

**Authors:** Cornelia Johanna Dorothy (Hanneke) Goense, Thuan-Huong P. Doan, Eneyi E. Kpokiri, Ymke J. Evers, Claudia S. Estcourt, Rik Crutzen, Jeffrey D. Klausner, Weiming Tang, Paula Baraitser, Christian J.P.A. Hoebe, Nicole H.T.M. Dukers-Muijrers

**Affiliations:** 1 Department of Social Medicine, Care and Public Health Research Institute (CAPHRI), Maastricht University, P.O. Box 616, Maastricht, 6200 MD the Netherlands; 2 Department of Sexual Health, Infectious Diseases and Environmental Health, Living Lab Public Health, Public Health Service South Limburg, Heerlen, Netherlands; 3 Department of Health Promotion, Care and Public Health Research Institute (CAPHRI), Maastricht University Medical Centre (MUMC+), Maastricht, Netherlands; 4 University of Southern California, Los Angeles, USA; 5 Clinical Research Department, Faculty of Infectious and Tropical Diseases, London School of Hygiene and Tropical Medicine, London, UK; 6 School of Health and Life Sciences, Glasgow Caledonian University, Glasgow, UK; 7 University of North Carolina at Chapel Hill, Project-China, Chapel Hill, NC USA; 8 Department of Sexual Health, King’s College Hospital NHS Foundation Trust, London, UK; 9 Department of Medical Microbiology, Infectious Diseases and Infection Prevention, Care and Public Health Research Institute (CAPHRI), Maastricht University Medical Centre+ (MUMC+), Maastricht, Netherlands

**Keywords:** Home care services, STI testing, HIV testing, Comprehensive care, Sexual health care, Realist review, Intervention, Key populations

## Abstract

This review identifies which elements of home-based comprehensive sexual health care (home-based CSH) impacted which key populations, under which circumstances. A realist review of studies focused on home-based CSH with at least self-sampling or self-testing HIV and additional sexual health care (e.g., treatment, counseling). Peer-reviewed quantitative and qualitative literature from PubMed, Embase, Cochrane Register of Controlled Trials, and PsycINFO published between February 2012 and February 2023 was examined. The PRISM framework was used to systematically assess the reach of key populations, effectiveness of the intervention, and effects on the adoption, implementation, and maintenance within routine sexual health care. Of 730 uniquely identified records, 93 were selected for extraction. Of these studies, 60% reported actual interventions and 40% described the acceptability and feasibility. Studies were mainly based in Europe or North America and were mostly targeted to MSM (59%; 55/93) (R). Overall, self-sampling or self-testing was highly acceptable across key populations. The effectiveness of most studies was (expected) increased HIV testing. Adoption of the home-based CSH was acceptable for care providers if linkage to care was available, even though a minority of studies reported adoption by care providers and implementation fidelity of the intervention. Most studies suggested maintenance of home-based CSH complementary to clinic-based care. Context and mechanisms were identified which may enhance implementation and maintenance of home-based CSH. When providing the individual with a choice of testing, clear instructions, and tailored dissemination successful uptake of STI and HIV testing may increase. For implementers perceived care and treatment benefits for clients may increase their willingness to implement home-based CSH. Therefore, home-based CSH may determine more accessible sexual health care and increased uptake of STI and HIV testing among key populations.

**Supplementary Information:**

The online version contains supplementary material available at 10.1007/s10461-024-04415-x.

## Background

Sexually transmitted infections (STI) such as *Chlamydia trachomatis* (CT), *Neisseria gonorrhoeae* (NG), Hepatitis B (HBV), Hepatitis C (HCV), syphilis, and human immunodeficiency virus (HIV) constitute a public health concern [1]. If STI or HIV are left undiagnosed and untreated, this could lead to serious adverse health consequences including acute illness, infertility, and long-term disability [2]. Regular testing followed by timely treatment is an effective strategy to improve clinical outcomes. Barriers to regular testing include social barriers such as expected HIV-related stigma and structural barriers such as costs or distance to the clinic [3, 4]. Home-based interventions, including self-(sampling) testing for HIV and STI, have gained urgency and popularity, especially during the last years of the COVID-19 pandemic [5]. Home-based interventions may lower commonly reported barriers and enhance personal autonomy thereby increasing accessibility and uptake of testing [6–8]. The latter could result in health benefits especially for key populations (i.e., populations that may be at elevated risk for HIV, for example men who have sex with men (MSM), sex workers, and transgender people). Home-based comprehensive sexual health care (hereafter: home-based CSH) includes either self-sampling or self-testing STI/HIV and additional sexual health care such as treatment or counseling. Self-sampling is when a person collects samples and sends them to a laboratory for testing, self-testing is when a person samples and interprets the test results themselves [9]. Self-sampled and clinician-collected samples provide comparable results for CT and NG testing, similar to the performances and usability of several HIV self-tests [10]. Therefore, the World Health Organisation (WHO) has recommended making self-sampling or self-testing STI and HIV available in addition to clinic-based sexual health services [8]. Earlier reviews about home-based CSH have mostly focused on its outcomes in terms of effectiveness and patient experiences of the care offered, with less attention to the implementation process in real-world settings [11–13]. The realist approach is especially relevant for understanding complex interventions, such as home-based and sexual health care [14]. Where systematic reviews usually aim to answer whether an intervention provides desired results, a realist review is particularly concerned with understanding why and how an intervention may or may not work, under which circumstances, and for which populations [15]. This realist review aimed to provide an overview of the mechanisms and contextual factors that determine what elements within home-based CSH impact which key populations under what circumstances. The Practical Robust Implementation and Sustainability Model (PRISM) was used as a structural guidance. Contextual domains, intervention description and outcomes such as reach, effectiveness, adoption, implementation, and maintenance assess the impact of public health interventions [16]. This realist review used existing literature to identify working mechanisms and outcomes, which are influenced by contextual factors such as different settings and populations, thereby providing valuable information for key populations, care providers, and policymakers.

## Methods

**Fig. 1:**
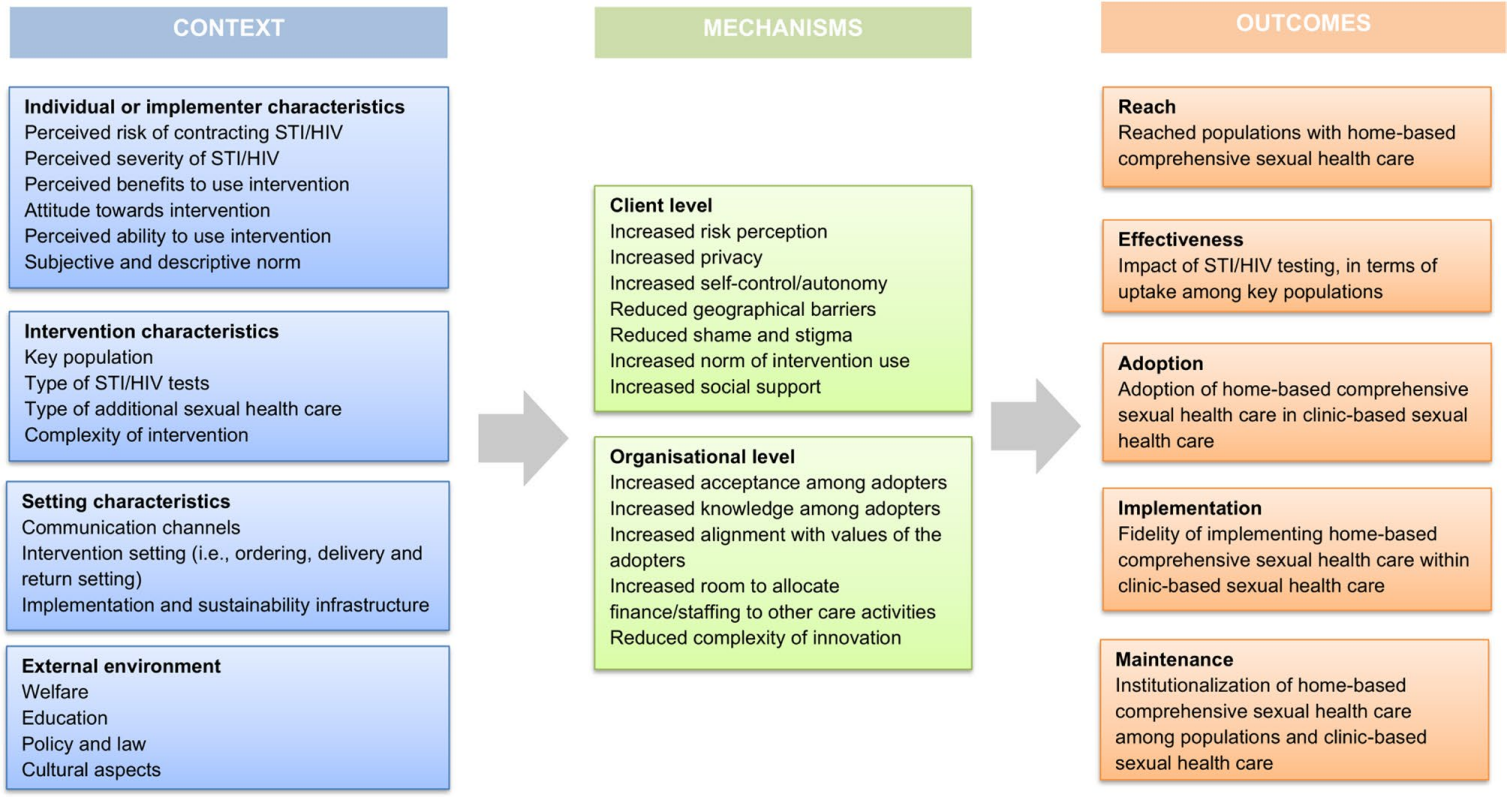
Initial program theory: theoretical framework of context, mechanisms, and outcomes

We conducted a realist review, which required the construction and refinement of an initial program theory. Figure 1 shows the initial program theory that assessed context, mechanisms, and outcomes. Contextual domains demonstrated characteristics of implementers, external environment (i.e., continent where intervention occurred, targeted population) and implementation and sustainability infrastructure (i.e., resources, adopter roles and monitoring). Intervention description examined a description of the different sorts home-based CSH and their content (type of testing, instructions and communication and dissemination). Outcomes were determined by reach(R), effectiveness(E), adoption(A), and implementation fidelity(I) elements of home-based CSH and how it affects the maintenance(M) of interventions [16]. We conducted the following iterative steps: (1) clarified research scope, (2) searched for relevant evidence, (3) appraised and extracted data, (4) synthesized evidence, and (5) evaluated findings [14]. This review is reported following the publication standards realist and meta-narrative evidence Syntheses (RAMESES) guidelines for realist synthesis [17]. This study is pre-registered in PROSPERO (CRD42023397383).

### Inclusion Criteria

Eligible interventions had a self-testing or self-sampling testing component. The intervention of home-based comprehensive sexual health care must consist of HIV testing alone or combined with other STI tests performed by the individual outside of a clinic location. In addition, the interventions should have other care components, such as sexual health counseling, partner notification, or linkage to treatment. Interventions could target any population at elevated risk for HIV infection, such as men who have sex with men, sex workers, or transgender people. We also included people living with HIV (PLWHIV), since they are at elevated risk for other STI. An exception on the inclusion criteria for HIV testing is applied for this population.

### Search Strategy

PubMed, Embase, PsycINFO, and Cochrane Central Register of Controlled Trials (CENTRAL) were searched [18]. The initial search aimed to explore initial key theories in the several interventions and refine inclusion criteria. The initial search was conducted in February 2022, and analysis and selection of articles was completed in June 2022 and covered literature from the past 10 years [see Supplementary information 1]. We adjusted the search strategy according to different terminology used to refer to home-based CSH in studies (i.e., telehealth, self-managed testing, home-testing). The first author conducted the initial search and together with the second and third authors performed independent title, abstract, and full-text screening. Literature was selected based on pre-specified inclusion and exclusion criteria [see Supplementary Information 2].

### Evidence Identification

**Fig. 2:**
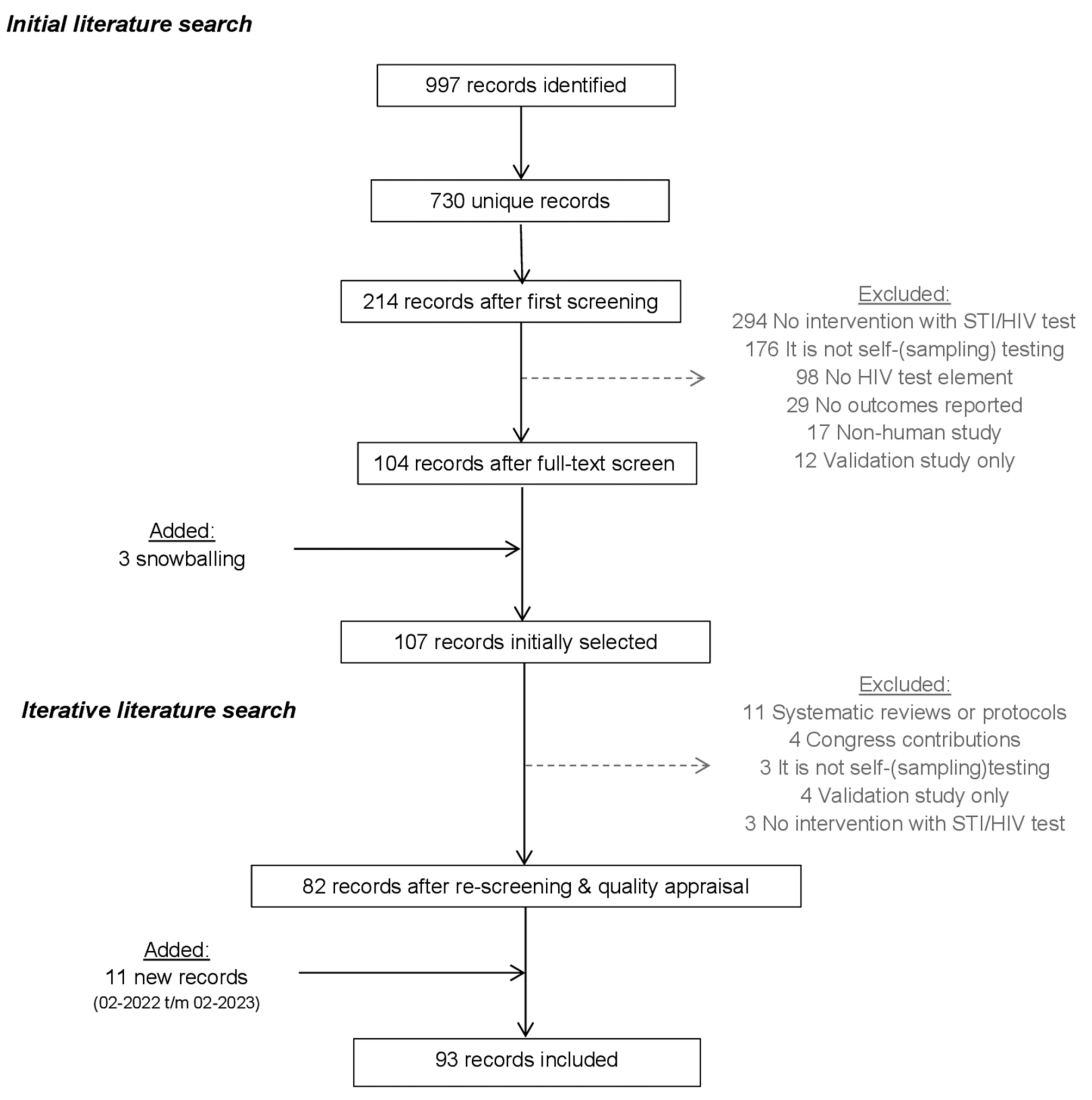
Flowchart of study search strategy through an iterative search process

The first three authors independently reviewed and analyzed a random selection of approximately 200 to 300 unique records each, all authors approved of the final selection. Figure 2 shows that from a total of 730 unique records identified, and after exclusion of 620 records, 107 studies (17%) had been initially selected for review. The main reasons for exclusion were ‘intervention did not include HIV testing’ or ‘HIV testing is not self-sampling or self-testing.’ Two reports were added to the selection using snowballing (i.e., referenced by included articles but not yet included in the search). An iterative selection excluded another 25 studies, mostly because they were systematic reviews or did not fit the research purpose. The final search added 11 studies from February 2022 to February 2023.

Of all selected studies, we identified the target population, N, continent where study took place, key findings and to which outcome the study contributed [see Supplementary Information 3]. Evidence identification was primarily conducted by the first three authors, the fourth and last author supervised this process.

### Quality Appraisal

The quality of the literature was determined by judging to what extent the selected literature contributes to the outcome of the study questions. The selection was appraised by following the mixed methods appraisal tool (MMAT) and additional questions were assessed such as (a) did the study describe constructs of the initial program theory (Fig. 1) (b) were intervention components described (c) was the context of the intervention described and (d) did the study describe the way the intervention affects outcomes (of reach, effectiveness, adoption, implementation, or maintenance) [19]. Studies were considered sufficient quality if clear research questions were reported, and the data allowed to address the research question. The first author conducted the quality appraisal which was randomly checked by the second and third author, inconsistencies were decided upon among the first three authors [see Supplementary Information 4]. All authors agreed with the overall selection procedure. The data extraction process is mainly conducted by the first author, other authors verified the process and provided feedback. Results were presented using the systematic framework of PRISM; herein the enhancing process factors that were identified based on evidence from all included studies, were grouped according to the level these factors acted upon, i.e., levels of on context (C), on mechanisms (M), and on outcomes (O).

## Results

A total of 93 studies were included that examined interventions of home-based CSH with STI and HIV self-sampling or self-testing and additional sexual health care [see Supplementary Information 4]. Most studies (60%; 56/93) described research with intervention components in randomized, non-randomized, quasi-experimental (qualitative and quantitative) evaluation studies. The other studies (40%; 37/93) solely described the acceptability or intention of key populations to potentially use an intervention (hereafter: acceptability studies). Evidence from 56 evaluation studies assessed intervention components in real-life settings. Results from 37 acceptability studies were additionally assessed for in-depth information.

### External Domains

#### Characteristics of Implementers

Implementers are considered the individuals who enable access to home-based CSH or handle the patient management of people who are using the intervention. Implementers were mainly care providers or counsellors. The context where home-based CSH was accessible was outside of a clinic setting, mostly for at-home use (39%; 22/56), or at the venue of recruitment (16%; 9/56).

#### Implementation and Sustainability Infrastructures

Implementation and sustainability infrastructures were not often described in detail, although some studies highlighted IT challenges. Expected pragmatic challenges were in data collection and reporting. Tailored programs may be required for implementing home-based care, although that could result in complex data management [20, 21]. Implementing a secured digital platform for data may improve access. A few studies described their challenges with digital applications such as missing links between test kits and participants, or survey errors [20, 22]. Digital applications for home-based CSH were mainly created for use in high-income countries and may be less applicable to lower-income countries [21].

#### Characteristics of the Intervention and Setting

Most included studies assessed HIV self-testing (54%; 30/56) or self-sampling HIV testing (32%; 18/56) interventions, and 16 (11%; 6/56) intervention studies were combined with self-sampling STI testing. Samples required for HIV testing were either blood or saliva. Blood-based samples were more accurate for syphilis, HBV, HCV, and HIV testing [23–25]. Blood was collected via finger-prick for dried-blood spot (DBS) or capillary collection (in a tube) or by a lancet in a vacuum system. Self-sampling testing for NG, CT required mainly swabs (i.e., urethral, pharyngeal, anorectal, vulvovaginal) and/or urine, there were no self-testing options available. The context of instructions to use the test kit were available before the actual testing and demonstrated by a care provider or study staff, or instructions were provided during testing with a video or written instructions. Several interventions did not specify the form in which instructions were provided. Various dissemination strategies were used in context to offer home-based CSH to key populations, and several venues were targeted. These include community venues, sex-on-premises, online services, or existing (location-based) sexual health care. Only a few studies elaborated on the method to design dissemination methods.

#### External Environment

In the context of the external environment for implementing home-based CSH, policy and culture around sexual health care vary greatly worldwide. There might be differences in external environment between high and low-and middle-income countries (LMIC). Whereas HIV is a worldwide public health concern, in LMIC the external environment structural possibilities (i.e., financing, services) may be limited [1]. Most studies included in this review were conducted in high-income countries such as within Europe (*n* = 30) or North America (*n* = 28), other studies were from LMIC.

#### Characteristics of Program Recipients

**Fig. 3:**
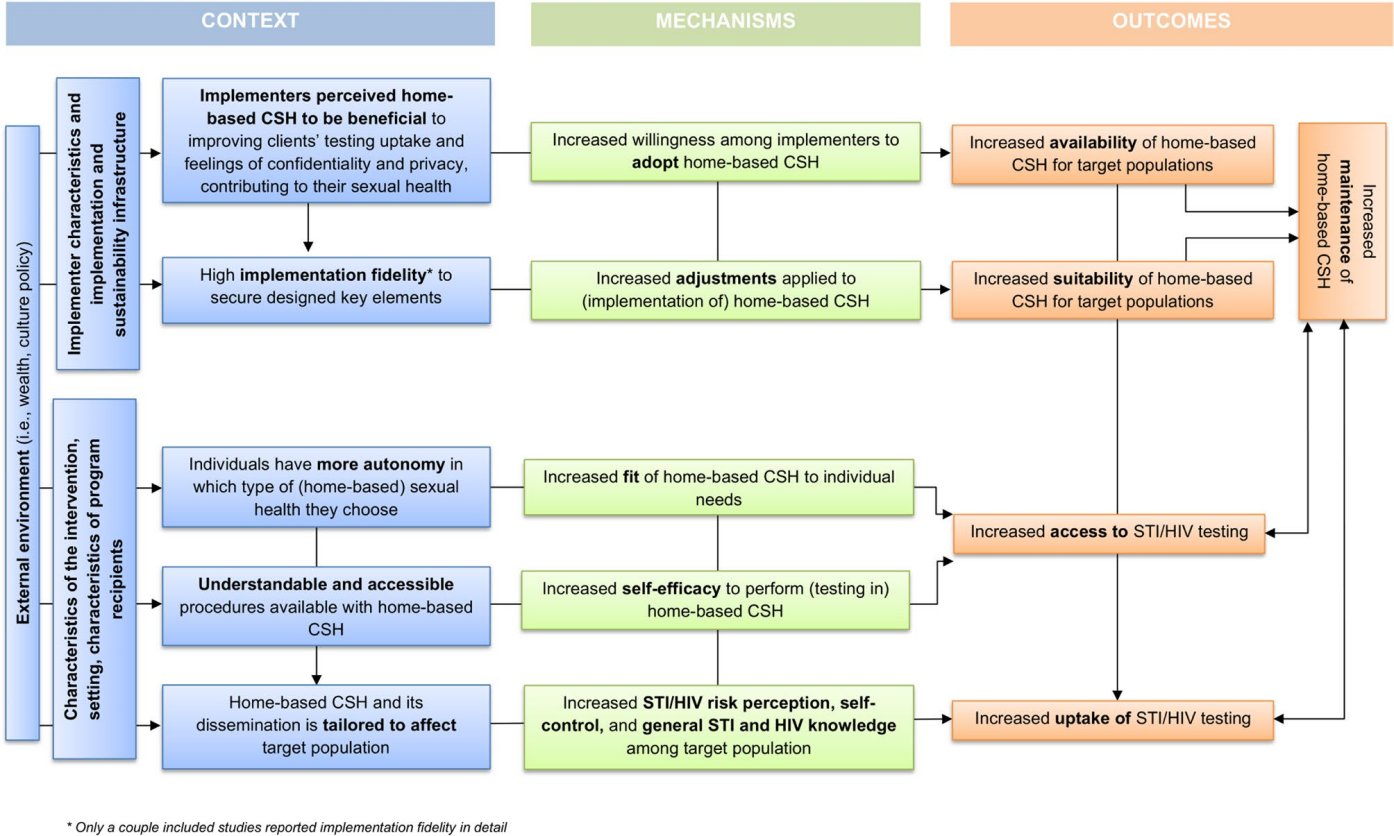
Context and mechanisms that enhance maintenance of home-based comprehensive sexual health care and uptake of STI/HIV testing

Although we included all target populations, most offers were focused on key populations MSM (*n* = 55), sex workers (*n* = 7), and transgender people (*n* = 6). All intervention studies focused on populations which were at elevated risk for HIV (except for PLWHIV). Figure 3 shows a summary of enhancing context elements which contribute to successfully implementing home-based CSH in practice and maintenance of its value for sexual health care of key populations in the long term. Elements were examined on the level of context, mechanisms, and how this contributes to outcomes.

### Reach

The type of self-sampling or self-testing is an important context element for the acceptance and use of home-based CSH. In a study among the general population of the United Kingdom, self-testing was mostly preferred, and self-sampling testing when the ability to complete the test within the laboratory [26]. Relevant mechanisms were described such as self-efficacy and knowledge of (correctly) performing the tests, acceptability of the offer. The use of a finger-prick was acceptable to people in key populations across most interventions. It was feasible and could be done successfully [27, 20]. Reported challenges were mainly a result of insufficient knowledge of correct performance [28–30]. Blood collection with a vacuum system was highly acceptable and feasible for sampling among PrEP-using MSM. They were confident of performing this without supervision at home [31]. Saliva-based sampling was also experienced as acceptable and mostly easy to perform [29, 32–36]. In addition, saliva-based samples had a higher return rate than blood samples [25]. Most studies were restricted to results of a single type of testing, either blood-based or saliva-based. However, interventions that assessed both, recommended that both self-testing and self-sampling HIV testing should be available to achieve an outcome of increased uptake [27, 37]. Clear instructions were reported to increase the mechanism of usability of self-(sampling) testing [28, 36, 38, 39]. Complementary (photo or animated) images of the puncture site could support correct performance (i.e., increased self-efficacy) [27]. Studies among African populations highlighted the importance of instructions in local languages [36, 40, 41]. Interventions in younger key populations reported high acceptability of online or mobile application instructions [22, 42, 43]. A couple studies used the intervention mapping framework to fit the communication messages, channels, and strategies to the needs of the key population [33, 44]. Other interventions only mentioned the communication modes (online or offline) [43, 45–47]. Online dissemination was mostly preferred by key populations when privacy concerns were related to clinic-based care [29, 37, 48, 49]. For key populations with inadequate access to digital technology, additional offline options (i.e., locations visited by key populations) should be available [50, 51]. Several studies demonstrated that online or peer-to-peer dissemination might be a promising strategy for reaching first-time testers [43, 47, 52–56]. For sex workers, self-tests were reportedly distributed through their workplaces (i.e., sex on premises, saunas, brothels), mobile units, and via online personal messages [42, 57, 58]. To recruit vulnerable key populations such as transgender people, online geotargeting was used. In addition, the costs of HIV self-(sampling) testing are mentioned as a barrier to testing uptake, and this is important to consider [59, 60]. Mechanisms such as accessible and tailored home-based CSH were applied in order to achieve increased uptake of STI and HIV testing among key populations.

### Effectiveness

The main reason organizations implement home-based CSH is to accomplish an outcome of increased HIV testing in the people who need it [6–8]. The effectiveness of an accessible care offer could thus be identified by the numbers of test uptake, HIV diagnosis, linkage to sexual health care, and prevention measures. Most studies demonstrated the identification of new HIV diagnoses. Overall, though HIV positivity rate varied between different contexts and populations, from 0.2% in a British study to 14.3% in a Chinese study among MSM [34, 61]. A substantial number of studies did not identify any new positive HIV results [26, 28, 32, 33, 35, 36, 22, 54, 62, 63]. Those studies were mainly based in Western continents (Europe, North America) and are focused on MSM. In studies that did not report positivity rates, the effectiveness of home-based care was attributed to the uptake of testing among at-risk and previously untested people and the return rate of test kits [52, 64–66]. Several interventions demonstrated high intention among populations to use home-based CSH again [35, 39, 22, 47, 67]. The mechanism were home-based CSH may fit the target group could be explained by a context of behavioral change of the individual, such as increased self-control, increased risk perception, and increased general knowledge of STI and HIV [35, 57, 63, 68]. Of 21 intervention studies that identified new HIV diagnoses, most of them also reported linkage to care such as confirmatory testing, linkage to treatment, post-test counseling, PrEP care, and partner notification. A few studies linked all new HIV infections successfully to care [60, 69]. Some studies reported referral, although it remained unknown whether clients had acted upon the referral to health care services [58, 69]. Other studies described loss to follow-up after positive HIV tests [46, 50, 61, 70]. Linkage to care in standard care is > 95%, and the linkage to care proportions of home-based care are comparable to outreach standards, i.e., within 75-100% [43]. There were 13 studies that did not describe which additional sexual health options were offered (e.g., linkage to treatment, counseling, partner notification).

### Adoption

Here, eleven studies examined adoption context by implementers and conditions for potential implementation in practice. Implementation of home-based CSH is needed for the availability and, thereby the accessibility and suitability of the offer (outcome). Most studies described experienced or perceived facilitators and barriers to adopting home-based CSH [20, 33, 35, 44, 48, 71]. Overall, adoption was acceptable according to implementers as a result of mechanisms such as increased access for clients to testing and increased confidentiality and privacy for clients. Implementers also mentioned the expected extended reach of vulnerable key populations and justification of clinical time (i.e., reduced, or reallocated workload) [44, 71]. One of the concerns of adopting home-based CSH was expected missed opportunities for appropriate sexual health prevention information and interventions (e.g., vaccinations), that could have been given when consultations take place in person [29, 30, 33, 71]. Other concerns were regarding following up on people with positive results when testing outside a clinic [35, 72].

### Implementation

Implementation fidelity indicates the context to which designed elements were used by implementers, as intended. Implementers, such as care providers, may have limited role tasks in carrying out the intervention since home-based CSH is designed for use by clients outside the clinic setting. Little information is available on the implementation fidelity of home-based interventions. One study demonstrated fidelity in the performance of the intervention. In one study, the performance fidelity of oral HIV self-testing was lower than expected since more than half of the participants had observed errors in their tests [73]. Another study described on an organizational level that a standardized script was recommended (i.e., a written version of all relevant elements) when introducing home-based CSH among care providers and their clients. Although the intervention was designed with these standardized elements, in practice tailoring was applied, which contributed to increasing the speed of processes [33]. A study from the initial literature search described adaptations to the intervention resulting in excluding syphilis testing from the home-based CSH [74]. They suggested considering a platform in implementation for at-home syphilis testing. Lastly, one study suggests simplification of intervention processes should contribute to implementation of home-based CSH, but without specifying the process simplification [60]. When implementation fidelity may be considered, it affects the adjustments made to the implementation of the intervention (mechanisms) and may therefore increase the outcome of suitable and accessible home-based CSH.

### Maintenance

The maintenance CSH assessed to what extent home-based CSH becomes an institutionalized part of routine sexual health care (outcome). Most studies suggested that home-based CSH may be offered complimentary to existing health care initiatives. For instance, a North American study evaluated the implementation of several oral HIV self-test interventions between 2018 and 2020, however, it did not examine its potential institutionalization within existing sexual health care [20]. One study demonstrated that a self-sampling service among MSM increased awareness and intention to test, two years after implementation [75]. From a user perspective, enhancing elements were identified among the included literature. The choice of either home-based or clinic-based STI and HIV testing, clear instructions, and tailoring of dissemination, which may contribute to the uptake of STI and HIV testing. Choice in the type of testing should increase the fit of the offer to individual needs. From the implementers’ perspective, few insights on home-based CSH were demonstrated. Even though adoption by implementers may be essential to successfully implement and maintain an intervention. In most cases, the long-term impact of most home-based CSH has not yet been examined, since it has been implemented in recent years.

## Discussion

This realist review assessed which elements of home-based comprehensive sexual health care (CSH) works for which key populations, and under which circumstances. A realist approach allowed for comparing key elements in a real-life context following the PRISM framework with reach, effectiveness, adoption, implementation, and maintenance outcomes. A recent systematic review of randomized controlled trials demonstrated a contribution to the uptake of HIV testing when offering self-testing instead of standard-of-care testing [76]. Several studies indicated a higher uptake of HIV testing as a result of implementing home-based CSH [47, 62]. However, another systematic review demonstrated growing inequality, with vulnerable groups less represented among home-based CSH users. Home-based CSH may not be suitable when people need further clinical evaluation in case of poor sexual health and or digital literacy or for vulnerable people [25]. Therefore, home-based CSH should be offered in addition to clinic-based sexual health care in order for people to have a choice in the type of testing and care they prefer [51]. Furthermore, an understandable and clear provision of test instructions could increase the self-efficacy of users. Previous studies identified that self-collected swabs and urine samples could be an alternative to swabs collected by a clinician [77]. Therefore, it is essential to offer clear instructions (i.e., written, illustrated, or video) to ensure correctly taken samples [78]. A recent study among MSM and transgender people found users preferred video instructions when using an HIV self-test. In addition, inaccuracies in the interpretation of HIV testing results were reduced, when using visualized instructions [79]. Moreover, previous studies suggested that if dissemination strategies are tailored, home-based CSH will be more accessible to key populations [41]. Qualitative evaluation of a self-collection program assessed that the use of multiple communication channels is preferred [80]. In addition, a previous literature review of oral HIV self-testing emphasizes making the offer fitting and accessible for the needs of the key population in terms of language, sexual health literacy, and culture [81]. In a previous study, care providers found the distribution of home-based CSH highly acceptable. However, there was concern about which care provider or health care organization should be responsible for managing clients when testing at home [82]. Other challenges for implementation may be the external environment and willingness of local policymakers. Although these contextual elements might affect implementation of home-based CSH information on local policy involvement lacks in studies. A previous review did highlight the collaboration between policymakers and epidemiologist to target those at elevated risk for HIV with self-testing [83]. On an organizational level, the cost-effectiveness of finding new STI and HIV infections is a facilitator to adopt home-based solutions [33, 42, 58]. However, at first, home-based CSH may have higher costs due to starting up processes required for implementation (e.g., collaboration with stakeholders, and monitoring) [84]. Another concern is the capability of proper performance of HIV self-testing by clients [82, 85]. A complete care package with self-testing and additional web-based information or counseling may improve the acceptability of home-based CSH by care providers [86]. In addition, simplification of tests should contribute to the ability of clients to perform their home-based testing accurately. Further, self-sampling testing (i.e., when clients send their samples to a laboratory) might offer care providers more control over clients’ test results and follow-up if necessary [26].

### Strengths and Limitations

To our knowledge, this is the first realist review that aimed to examine enhancing elements of global home-based CSH. In addition, the realist approach seemed appropriate for assessing this complex intervention. However, a high number of studies had different definitions for home-based CSH and could therefore not be compared properly in such syntheses. Terms such as telehealth, eHealth, and self-managed care, among other terminology, are used interchangeably to refer to different elements within home-based CSH. The WHO has called for clear and transparent definitions of digital health and self-care [87]. Earlier, the nomenclature was determined for self-testing and self-sampling testing [9]. Universal nomenclature in scientific articles may contribute to increased comparability and transparency of studies. Furthermore, a limited number of studies were included from implementation and maintenance perspectives. This could result in missing important conditions for implementation in the realist setting and the long-term impact of home-based CSH. As most included studies focused on MSM, information is lacking for other key populations, a common occurrence [76, 88, 89]. Therefore, future research should consider sampling key populations such as migrants, transgender people, and sex workers.

### Implications

Home-based comprehensive sexual health services should complement clinic-based care to provide individuals with options for their preferred testing and care methods. Clear and understandable test instructions, as well as simplifying the tests can help clients perform home-based testing accurately. An example would be a comprehensive home-based care package, including self-(sampling) testing and web-based information and counseling. Accessibility can be improved by tailoring services to the key population’s language, sexual health literacy, and cultural needs. For implementers, the cost-effectiveness and maintaining control over detecting new STI and HIV infections supports the adoption of home-based solutions. For implementers, the cost-effectiveness and ability to maintain control over detecting new STI and HIV infections support the adoption of home-based solutions.

## Conclusions

Home-based comprehensive sexual health care (home-based CSH) interventions have been implemented globally in recent years. This realist review aimed to identify which elements within home-based CSH works for which key populations under which circumstances. PRISM elements for enhancing the reach, effectiveness, adoption, and implementation of interventions were identified; choice of testing to fit individual needs, provision of clear instructions, tailored dissemination, increased individual determinants for successful uptake, and perceived care and treatment benefits for clients. Considering these elements within home-based CSH may result in maintenance in regular sexual health care. Therefore, accessibility of sexual health care may be increased for key populations, affecting uptake of testing and care.

## Electronic Supplementary Material

Below is the link to the electronic supplementary material.

Supplementary Material 1

Supplementary Material 2

Supplementary Material 3

Supplementary Material 4

## Data Availability

All data generated or analyzed during this study are included in this published article [and its supplementary information files].
