# Supplementary material for: Understanding Practical, Robust Implementation and Sustainability of Home-based Comprehensive Sexual Health Care: A Realist Review
Source: AIDS Behav. 2024 Jul 4;28(10):3338–49. doi: 10.1007/s10461-024-04415-x (PMC11427609; doi:10.1007/s10461-024-04415-x)
Supplement: Supplementary file 2 — Supplementary Material 2 [file 10461_2024_4415_MOESM2_ESM.pdf]

Supplementary Information 2. Inclusion and exclusion criteria

Inclusion criteria:

- Date of publication: up to 10 years ago
- Language: English
- Study design: ALL
- Population: primarily focused on key populations (e.g., young individuals, migrants, sex workers, MSM, transgender people, gender diverse people, bisexual and homosexual men, swingers, PWID, PLWHIV)
- Intervention: home-based comprehensive sexual health care with (online) self-sampling for HIV and/or STI testing and AT LEAST one of these care elements:
    - Results provision
    - Treatment
    - Partner notification
    - Health promotion/prevention
    - Retesting/repeat testing
- Studies should report AT LEAST one of these **outcomes** and/or **constructs*** (Following PRISM outcomes)

Exclusion criteria:

- Non-human studies
- Validation studies with only test related results (sensitivity, specificity)
- Intervention has no HIV testing component
    - Unless key population is HIV positive

*Table of constructs related to PRISM outcomes: reach, effectiveness, adoption, implementation, and maintenance.

PRISM	Answer to…	Signal words
**Reach**	"How do I reach the targeted population?"	Reach<br>Target<br>Number of …<br>Proportion of …<br>Represent
**Effectiveness**	"How do I know my intervention is effective?"	Efficacy<br>Impact<br>Effects /Affect<br>Effective(ness)<br>Influence<br>Outcome<br>Factors<br>Predictors
**Adoption**	"How do I develop organizational support to deliver my intervention?"	Adoption<br>Setting<br>Deliver<br>Support<br>Barriers<br>Facilitators

**Implementation**	“How do I ensure the intervention is delivered properly?”	Implementation<br>Fidelity<br>Protocols
**Maintenance**	“How do I incorporate the intervention so that it is delivered over the long term?”	Maintenance<br>Policy<br>Incorporation<br>Long term<br>Follow-up
